# Overlooked aspects of scaling enzyme activity through abundance across tissues and individuals: Insights from *k*_cat_ measurements in matched liver and intestinal samples

**DOI:** 10.1016/j.dmd.2025.100229

**Published:** 2025-12-29

**Authors:** Zubida M. Al-Majdoub, Jill Barber, Amin Rostami-Hodjegan, Aleksandra Galetin, Daniel Scotcher

**Affiliations:** 1 Centre for Applied Pharmacokinetic Research, University of Manchester, Manchester, United Kingdom; 2 Certara Predictive Technologies, Sheffield, United Kingdom

**Keywords:** Drug metabolism, Liver, Intestine, Cytochrome P450, UDP-glucuronosyltransferase, Physiologically based pharmacokinetic modeling

## Abstract

Prediction of human intestinal metabolism within physiologically based pharmacokinetic models is now well established within drug development. Extrapolation of in vitro kinetic parameters accounts for differences in abundance between different in vitro systems and tissues. The existing data assume that the activity of CYP3A4 is consistent between the intestine and liver once adjusted for its tissue-specific expression level. However, the validity of this assumption for other enzymes and other tissues remains uncertain. In the current study, indicators of "activity per unit of enzyme," namely, turnover number (*k*_cat_) or specificity constant (*k*_sp_), were measured for 7 enzymes (CYP2C9, CYP2C19, CYP2D6, CYP3A4, UGT1A6, UGT2B7, and UGT2B17) in microsomes prepared from 4 paired (same donor) intestine and liver tissue samples. After excluding 1 donor with low intestinal activity, the intestinal *k*_cat_ and *k*_sp_ for the studied CYPs were within 2-fold of the liver values, with the exception of 1 donor with 4-fold lower CYP2D6 *k*_cat_ in the intestine compared with the liver. Conversely, the UGT1A1 *k*_sp_ and UGT2B7 *k*_cat_ were 5-fold and 7-fold higher in intestinal microsomes compared with liver microsomes, respectively. Trends in interdonor variability in *k*_cat_ were noted and require further evaluation in a larger set of donors. The current paradigm of extrapolation of hepatic metabolism data to predict in vivo first-pass metabolism in the intestine using tissue abundances appears to be valid for CYPs but should be approached with caution when predicting intestinal glucuronidation.

**Significance Statement:**

This study assessed whether hepatic metabolism data can predict intestinal metabolism in physiologically based pharmacokinetic models by comparing enzyme abundance and activity in matched liver and intestine microsomes from 4 donors. Seven key drug-metabolizing enzymes were quantified. While CYP-mediated intestinal metabolism could generally be predicted from liver data after adjusting for tissue abundance, caution is warranted for enzymes involved in intestinal glucuronidation, where assumptions of equivalent activity across tissues may not hold.

## Graphical abstract

**Figure:**
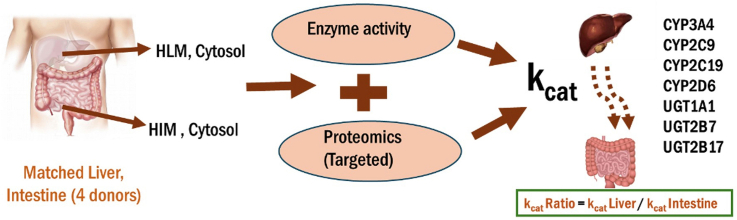

## 1 Introduction

Simulation of variability in pharmacokinetics using physiologically based pharmacokinetic (PBPK) models requires generation of virtual subjects, each with unique demographic, anatomical, and physiological characteristics (ie, “system data”).^1^ Some of the system data (eg, enzyme/transporter abundance) also inform some of the physiological scaling factors within the in vitro-in vivo extrapolation (IVIVE) paradigm to predict in vivo pharmacokinetics. The IVIVE of in vitro unbound intrinsic clearance (CL_int,u,in vitro_) measured using pooled human liver microsomes (HLMs) (or hepatocytes, analogously) applies scaling factors such as microsomal protein per gram liver and average total liver weight.^2^^,^^3^ These scaling factors can consider covariates like age and body weight^4^^,^^5^ to individualize predictions of in vivo CL_int,u_ (CL_int,u in vivo_). If sufficient reaction phenotyping has been performed (eg, using specific enzyme inhibitors), prediction of CL_int,u,in vivo_ can be further individualized by accounting for a virtual subject’s enzyme abundance (pmol/mg protein). PBPK simulations typically account for interindividual variability of CL_int,u,in vivo_ due to factors such as liver weight, enzyme abundance, and genotype. However, a common assumption is that the turnover number, representing the activity per unit of enzyme (*k*_cat_; nmol/min per pmol enzyme), or specificity constant (*k*_sp_ = *k*_cat_/*K*_M_ [Michaelis constant]; *μ*L/min per pmol enzyme; also termed kinetic efficiency) does not vary between individuals of the same genotype.

The extrapolation of enzyme kinetic data from various in vitro systems has been assessed for differences in specific activity per enzyme abundance, and the requirement for an intersystem extrapolation factor has been highlighted.^6^ However, very few studies have investigated specific activity per unit of enzyme using robust quantitative assays for abundance as well as specific enzyme probes. Our previous study suggested minor variability in CYP2B6 *k*_cat_ (CV ∼ 26% for subjects with the same genotype) compared with CYP2B6 abundance variability.^7^ Prediction of intestinal first-pass metabolism can follow a similar IVIVE-PBPK approach as used for the liver, by measuring enzyme activity using pooled human intestinal microsomes (HIMs) to obtain CL_int,u,in vitro_.^8^, ^9^, ^10^, ^11^ However, HIMs are generally less accessible than HLMs owing to limited high-quality tissue samples and a more onerous preparation process that may affect enzyme activity.^9^^,^^12^ In addition, enzyme expression varies across intestinal regions (eg, duodenum, ileum, and jejunum),^13^ which can confound data interpretation. A more pragmatic approach to predicting CL_int,u,in vivo_ in the small intestine is to obtain *k*_cat_ or *k*_sp_ using HLM with reaction phenotyping and then perform IVIVE scaling using intestinal system data.^8^^,^^14^ These data include enzyme abundance in enterocytes and total intestinal microsomal protein (or segment of intestine) and are established for healthy populations.^10^^,^^15^ This more pragmatic approach to prediction of intestinal CL_int,u,in vivo_ relies on the assumption that interorgan differences in drug metabolism can be accounted for solely by differences in protein abundance, and hence that the *k*_cat_ (or *k*_sp_) values are the same in the intestine as in the liver. However, this assumption has only been evaluated for CYP3A4.^8^ Available literature data to support this assumption for other enzymes and for other interorgan comparisons beyond liver and intestine (eg, liver and kidney) are currently sparse. Data from nonmatching tissue donors (where confounding effects of genotype differences may not be ruled out) and the lack of studies investigating *k*_cat_ of several enzymes between tissues from the same donors (Supplemental Table 1) are key limiting factors. In addition, intestinal and liver microsomal data (from same animals) for dog Cyp2b11 and Cyp3a12 substrates did not support an organ-independent *k*_cat_.^16^ Given these uncertainties, the assumption of tissue independent *k*_cat_ within IVIVE and PBPK modeling requires experimental validation.

The aim of this research was to test whether the *k*_cat_ of drug-metabolizing enzymes is tissue independent. To investigate this hypothesis, *k*_cat_ was measured in human liver, intestinal, and kidney microsomes for a panel of CYP and UDP-glucuronosyltransferase (UGT) enzymes. The enzymes investigated, CYP2C9, CYP2C19, CYP2D6, CYP3A4, UGT1A1, UGT1A9, UGT2B7, and UGT2B17 were chosen based on their clinically significant roles in drug metabolism, their relevant expression in the tissues studied, and the availability of well established probe substrates. These enzymes collectively account for the metabolism of a large proportion of clinically used drugs and are representative of different expression patterns across the liver, intestine, and kidney. Initially, activity and quantitative proteomics experiments were conducted using pooled liver, intestinal, and kidney microsomes. Next, *k*_cat_ was measured in matched liver and intestinal microsomes from 4 individual donors (ie, liver and intestine coming from the same donor). In addition to metabolic enzymes, the expression of CYP accessory proteins, such as cytochrome b_5_, NADPH-cytochrome P450 reductase, and NADH-cytochrome b_5_ reductase 3, was quantified in the same matched donors.

## 2 Materials and methods

### 2.1 CYP3A4 literature abundance data

Meta-analysis of literature abundance data used the PubMed database (https://www.ncbi.nlm.nih.gov/pubmed/ [2019–2020]) to identify relevant literature reporting CYP3A4 (pmol/mg protein) using relevant methods (eg, liquid chromatography-mass spectrometry [LC-MS] and Western blot), using appropriate keywords (eg, “CYP3A4,” “abundance”). Searches were species and tissue-specific for human liver, intestinal, and kidney microsomes; keywords included synonyms for these tissues (eg, “hepatic,” “gut,” and “renal”). The selection criteria for the studies included in our analysis for *k*_cat_ calculation were carefully defined to ensure the robustness and relevance of the data. A key consideration was tissue preparation methods, with studies included only if they used detailed and consistent tissue processing techniques. The choice of reagents had to be similar across all studies, and selected studies had a digestion length sufficient to achieve comprehensive protein breakdown and maximize peptide recovery for subsequent analyses (>20 hours). Moreover, studies were selected based on a sufficient sample size (>10 donors). The racial background of the study participants was also considered. Ultimately, these stringent selection criteria led to the inclusion of 3 studies for hepatic CYP3A4 abundance and 1 study for intestinal CYP3A4 abundance, providing data sets for the calculation of the weighted mean enzyme abundance for each tissue (eq. 1).

(1) $${\bar{x}}_{\omega }=\frac{\sum_{i=1}^{n}\omega_{i}{\cdot x}_{i}\;}{\sum_{i=1}^{n}\omega_{i}\;}$$

where ${\bar{x}}_{\omega }$ is the weighted mean abundance, $x_{i}$ represents the mean individual abundance values from each study, $\omega_{i}$ represents the corresponding weights for each abundance value by the number of donors in each study, and *n* is the total number of studies.

### 2.2 Chemicals and reagents

All chemicals and solvents (high-pressure liquid chromatography grade) were purchased from Sigma-Aldrich unless otherwise stated. Lysyl endopeptidase and Trypsin (sequencing grade) were obtained from Wako and Roche Applied Sciences, respectively. Bovine serum albumin (BSA) was obtained from Thermo Fisher Scientific. Stable isotope-labeled MetCAT (QconCAT standard) was supplied by PolyQuant GmbH (http://www.polyquant.com/). Non-naturally occurring peptide standard (light peptide) used for the quantification of MetCAT (QconCAT) was purchased from Cambridge Peptides.

Pooled HLM (BD Gentest, Corning, and BioIVT; lot numbers 36170 [33 donors], 38289 [150 donors], 38290 [150 donors], 88114 [50 donors], and BRE [150 donors]), pooled human kidney microsomes (HKM) (XenoTech; lot numbers 1710160 [8 donors], 1410120 [13 donors]), and pooled HIM (XenoTech; lot numbers 1110396 [13 donors], 1610314 [15 donors], 610108 [10 donors], 0710352 [18 donors], and BioIVT; lot GYC [6 donors]) were available from the Centre for Applied Pharmacokinetic Research historical sample stocks (stored at –80 °C for >10 years), except for BRE, GYC, 1710160, and 1610314 that were purchased for the current research. Individual human liver and matched intestinal microsomes from 4 donors (Table 1) were purchased from BioIVT. HIM fractions were prepared using the elution method.

**Table 1:** Donor characteristics for individual human liver and intestinal matched-donor microsomes

Donor ID	Sex and/or Gender	Ethnicity	Age y	BMI	Alcohol/Tobacco
D1	Male	Caucasian	58	39.1	No
D2	Male	Hispanic	56	30.6	Light
D3	Female	African American	42	34.9	Heavy (tobacco)
D4	Male	Caucasian	57	23.2	Heavy (both)

BMI, body mass index.

### 2.3 Functional activity assays

Probe drug substrates were incubated with HLM, HIM, and HKM to characterize the functional activity of CYPs and UGTs in these samples. Preliminary experiments were performed using pooled microsomes to establish optimal experimental conditions, which were then applied in the final experiments performed with individual matched-donor intestine and liver microsomes. Because of limitations in sample availability for certain lots, not all probes were assayed in every microsomal lot. Substrate depletion assays were performed for diclofenac (CYP2C9), ezetimibe (UGT1A1), propofol (UGT1A9; pooled samples only), and gemfibrozil (UGT2B7; pooled samples only) using the method described previously.^11^^,^^17^ Metabolite formation assays were performed for *S*-mephenytoin 4-hydroxylation (CYP2C19), dextromethorphan *O*-demethylation (CYP2D6), testosterone 6-*β*-hydroxylation (CYP3A), gemfibrozil glucuronidation (UGT2B7; individual donor samples only due to low sensitivity of the substrate depletion assay), and testosterone glucuronidation (UGT2B17). Because of the limited availability of microsomes, metabolite formation assays were conducted at a single high (above *K*_m_) substrate concentration to approximate apparent maximum velocity of the enzyme reaction (*V*_max_; pmol/min per mg protein). Deferipone (UGT1A6) substrate depletion was also explored but not included in the final data set because of low activity in pooled intestinal microsomes. Substrate and microsomal protein concentrations, incubation time, and BSA concentrations (for UGT substrates only) were selected to ensure sufficient assay sensitivity, and for measurement of first-order or zero-order reaction rates for substrate depletion or metabolite formation assays, respectively, as listed in Supplemental Table 2. Compound stock solutions were prepared in DMSO (with a maximum final assay concentration of 0.25%), except for *S*-mephenytoin, which required acetonitrile (final concentration 0.25%) to obtain sufficient solubility.

For CYP-mediated reactions, the compounds were preincubated at 37 °C with human liver or intestinal microsomes in 0.1 M phosphate buffer at pH 7.4. Reactions were initiated by the addition of NADPH, with a final cofactor concentration of 1 mM. UGT-mediated reactions were performed using 0.1 M phosphate buffer at pH 7.1 containing 3.45 mM MgCl_2_, 1.15 mM EDTA, 115 *μ*M saccharic acid lactone, and 1% or 2% BSA (see Supplemental Table 2). Human liver, kidney, or intestine microsomes were activated by incubation with alamethicin (50 *μ*g/mg protein) on ice for 15 minutes.^11^^,^^18^ Compounds were preincubated at 37 °C with activated microsomes for 5 minutes, after which the reactions were initiated by adding uridine 5′-diphosphoglucuronic acid at a final cofactor concentration of 5 mM. All reactions were terminated at specified timepoints by quenching with an excess of acetonitrile containing appropriate internal standard.

The quenched samples were then centrifuged and analyzed by LC-MS/MS. For metabolite formation assays, calibration curves of the respective metabolite standards were prepared using pooled HLM as a matrix and analyzed using LC-MS/MS to enable metabolite concentration quantification. Assays were performed in duplicate or triplicate, depending on sample availability, with a single no-cofactor control included to identify potential nonspecific instability or loss to binding.

### 2.4 Sample preparation for proteomics

Protein content in the HLM, HIM, and HKM pooled samples was estimated using Bradford assay (ThermoFisher Scientific). Approximately 0.40 *μ*g of stable isotope (^13^C)-labeled concatenated standard (QconCAT/MetCAT) was spiked into 50 *μ*g of each pooled sample and each individual sample as internal standard for the quantification of CYPs and UGTs.^19^^,^^20^ In addition, 0.126 *μ*g of BSA was spiked as a label-free exogenous protein standard for use in a global analysis. Sample preparation was performed using a filter-aided sample preparation approach,^21^ as previously described,^22^ with minor modifications detailed in the Supplemental Material (under Section 1).

### 2.5 Targeted LC-MS/MS analysis

Microsomal proteins from pooled and individual matched samples were analyzed by LC-MS/MS using a thermo rapid separation (RS) LC system consisting of an NCP3200RS nano pump, WPS3000TPS autosampler and TCC3000RS column oven configured with buffer A as 0.1% formic acid in water and buffer B as 0.1% formic acid in acetonitrile. An injection volume of 2 *μ*L was loaded into the end of a 5 *μ*L loop and reversed flushed onto the analytical column (Waters nanoEase M/Z Peptide charged surface hybrid C18 column, 130 Å, 1.7 *μ*m, 75 *μ*m × 250 mm) kept at 35 °C at a flow rate of 300 nL/min with an initial pulse of 500 nL/min for 0.1 minutes to rapidly repressurize the column. The separation consisted of a multistage gradient of 1% B to 6% B over 3 minutes, 6% B to 18% B over 67 minutes, 18% B to 29% B over 9 minutes and 29% B to 65% B over 1 minutes before washing for 6 minutes at 65% B and dropping down to 2% B in 1 minutes. The complete method time was 120 minutes. The analytical column was connected to a Thermo Exploris 480 mass spectrometry system via a Thermo nanospray Flex ion source via a 20-*μ*m inner diameter fused silica capillary. The capillary was connected to a fused silica spray tip with an outer diameter of 360 *μ*m, an inner diameter of 20 *μ*m, a tip orifice of 10 *μ*m, and a length of 63.5 mm (New Objective Silica Tip FS360-20-10-N20-6.35CT) via a butt-to-butt connection in a steel union using a custom-made gold frit (Agar Scientific AGG2440A) to provide the electrical connection. The nanospray voltage was set at 1900 V, and the ion transfer tube temperature at 275 °C.

### 2.6 Data acquisition, analysis, and protein quantification for targeted analysis

Data were acquired in retention time window mode, with an expected peak width of 15 seconds, and no full MS data were acquired. The target list of MetCAT peptides was imported into the method via an Excel .csv file, which included their previously determined retention times, with a retention time window of 4 minutes. Fragmentation spectra were acquired with a resolution of 15,000 with a normalized collision energy of 30%, the automatic gain control target set to standard, and a max fill time of 100 ms for a single microscan. All data were collected in profile mode. Skyline version 22.2 software (MacCross Lab Software) was used for the generation of the abundance data, as previously described.^22^ A list of the peptides that constitute the MetCAT used in this study is presented in Supplemental Table 3.

### 2.7 Global proteomic analysis of CYP accessory proteins using LC-MS/MS

The global analysis was conducted with Progenesis QI 4.0 (Nonlinear Dynamics).^23^ Cytochrome b_5_ type A and type B (CYB5A and CYB5B), NADPH-cytochrome P450 reductase, and NADH-cytochrome b_5_ reductase 3 were measured. The Centre for Applied Pharmacokinetic Research database, based on the Uniprot Human Protein fasta file (https://www.uniprot.org/proteomes/UP000005640), was used to determine the protein accessions. The database includes 21,520 proteins. The razor used for data analysis was described in our previous publication.^24^ The selection of peptides was according to previously published criteria.^25^ Quantification was performed using the averaged intensity of the 3 most abundant unique peptides (Supplemental Table 4) based on the acquired MS/MS data for the protein of interest, and the standard protein (BSA) was selected and used as an internal standard to enable label-free quantification of the liver, intestinal, and kidney proteins.^26^

### 2.8 Metabolism data analysis

In vitro metabolic rate constants (*k*; min^–1^) for microsomal substrate depletion assays were determined by linear regression of the natural log-transformed fraction of substrate remaining versus time, using the LINEST function in Microsoft Excel. The rate constant *k* corresponds to the negative of the estimated slope. Intrinsic clearance (CL_int_; *μ*L/min per mg protein) was then calculated from the rate constant *k* and the microsomal protein concentration, as previously reported.^17^ For metabolite formation assays, data were visually assessed to select the initial linear phase, from which metabolite formation rates were derived. The assay was performed at substrate concentrations exceeding the respective *K*_m_ (Supplemental Table 2), allowing determination of *V*_max_ by linear regression of metabolite formation versus time. In addition, CYP3A4 functional activity (measured as the rate of 6-*β*-hydroxytestosterone formation) data for various pooled human liver and intestinal microsomes (HLM: 36170, 88114, 38290, 38289, and BRE. HIM: 1110396, 610108, 1610314, 0710352, and GYC) were extracted from supplier product information sheets (BD Gentest, Corning, and XenoTech). The turnover number (*k*_cat_; pmol/min per pmol enzyme) was calculated using eq 2.

(2) $$k_{\text{cat}}=\frac{V_{\max}}{\text{Abundance}}$$

where *V*_max_ is the maximum velocity of the enzyme reaction (pmol/min per mg microsomal protein), and abundance refers to the abundance of the enzyme (pmol/mg microsomal protein).

*V*_max_ data were not available; the specificity constant (*k*_sp_; *μ*L/min per pmol enzyme) was calculated (eq. 3) as a surrogate for *k*_cat_.

(3) $$k_{\text{sp}}=\frac{{\text{CL}}_{\mathrm{int}}}{\text{Abundance}}$$

where CL_int_ is the microsomal intrinsic clearance (*μ*L/min per mg microsomal protein).

To investigate the impact of the source of proteomics abundance data on estimated *k*_cat_ tissue ratios, CYP3A4 *k*_cat_ for the liver and intestine were calculated (eq. 2) using our published intestinal abundance^27^ and either (1) literature hepatic CYP3A4 abundance weighted mean data from several studies using similar methodology to that in the paper by Couto et al^23^ (Supplemental Table 5), or (2) the average hepatic CYP3A4 abundance from our laboratory.^28^ These *k*_cat_ values were then compared with those calculated using CYP3A4 abundance measured in the specific lot of pooled tissue microsomes.

The *k*_cat_ ratio for 2 tissues were calculated using eq. 4

(4) $$k_{\text{cat}}\;\text{ratio}=\frac{k_{\text{cat},\text{tissue}\;b}}{k_{\text{cat},\text{tissue}\;a}}$$

where *k*_cat,tissue__*a*_ or *k*_cat,tissue__*b*_ may refer to the *k*_cat_ for 2 different tissues (eq. 2), or *k*_sp_ for 2 different tissues (eq. 3). Nonspecific binding was assumed to be equivalent between HLM, HIM, and HKM as the assay protein concentrations were the same.^29^

### 2.9 Statistical analysis

Statistical analysis of the data was carried out, and graphs were created using GraphPad Prism version 9.0 and Microsoft Excel 2016. Data were presented as mean and SD. CV was used to describe variability. Linear regression was used to determine in vitro metabolic rate constants (*k*; min^–1^).

## 3 Results

### 3.1 CYP and UGT activities in pooled hepatic, intestinal, and kidney microsomes

In vitro enzyme activity was measured in pooled HLM, HIM, and HKM for probes of CYP2C9, CYP2C19, CYP2D6, UGT1A1, UGT1A6, UGT1A9, UGT2B7, and UGT2B17 to evaluate tissue-specific *k*_cat_ values and assay conditions for subsequent assays with microsomes prepared from tissues of individual donors (Supplemental Tables 6–9). For CYP3A4, testosterone 6*β*-hydroxylation activity data were collated from the vendors’ product information sheets (Supplemental Table 10). Initial comparison used activity or clearance data expressed per of microsomal protein, which were not corrected for differences in enzyme abundance between the liver, intestine, and kidney. The liver pooled microsomes exhibited the highest CL_int_ (Supplemental Table 6) and metabolite formation rates (Supplemental Table 7). Diclofenac CL_int_/mg protein (CYP2C9) in liver microsomes was 20- to 60-fold higher compared with the intestine, and 30- to 190-fold higher than kidney microsomes. Ezetimibe CL_int_/mg protein (UGT1A1) was 36-fold higher in the pooled liver microsomes than in the intestine (Supplemental Table 6). Formation rate of 6-*β*-hydroxytestosterone/mg protein (CYP3A4) was 36-fold higher in the pooled liver microsomes than in intestine. Minimal depletion of deferiprone (UGT1A6) was observed in pooled HIM, whereas propofol depletion (UGT1A9) in the presence of the cofactor uridine 5′-diphosphoglucuronic acid was similar to the no-cofactor control in pooled HLM and HIM. Therefore, these probes/enzymes were not investigated in the individual matched-donor microsomal samples.

### 3.2 Abundance of enzymes in pooled liver, intestinal, and kidney microsomes

The abundance levels of enzymes (CYP3A4, CYP2C9, CYP2C19, UGT1A1, UGT1A3, UGT1A4, UGT1A6, UGT1A9, and UGT2B7) were measured in pooled HLM (*n* = 2), HIM (*n* = 2), and HKM (*n* = 2) with targeted proteomic analysis (Supplemental Tables 11–13). Substantially higher protein abundance was apparent in the pooled liver microsomes compared with intestinal samples particularly for CYP enzymes. CYP3A4, the most abundant enzyme, was 5- to 9-fold higher than in the intestine, whereas CYP2C9 had ≥10-fold lower abundance in the intestine compared with liver. UGT2B7 abundance was 3- to 26-fold higher in the liver compared with the intestine; in contrast, UGT2B17 had similar abundance in the intestine (11.7 pmol/mg protein) and liver microsomes (8.9 pmol/mg protein). UGT1A9 showed markedly lower expression in the intestinal microsomes (0.2 pmol/mg protein) compared with the liver (15.7–131.0 pmol/mg protein) and kidney (61.6 and 90.3 pmol/mg protein). Among all enzymes, CYP3A4 protein was found to be the most abundant in the intestinal microsomes (mean 25.1 pmol/mg protein; *n* = 5 pools), followed by UGT2B17 (11.7 pmol/mg protein; *n* = 1), UGT1A1 (9.4 pmol/mg protein, *n* = 3 pools), and UGT2B7 (mean 5.7 pmol/mg protein; *n* = 2). In HKM, the 2 most abundant enzymes were UGT1A9 (mean 146 pmol/mg protein; *n* = 2 pools) and UGT2B7 (mean 76 pmol/mg protein; *n* = 2).

### 3.3 Calculation of k_cat_ in different tissues using data from pooled samples

**Fig. 1:**
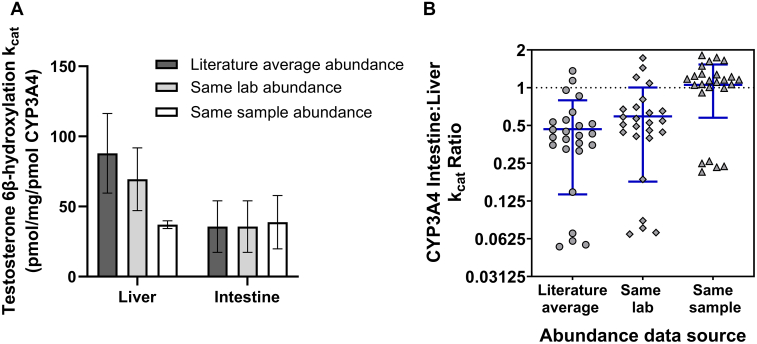
Impact of CYP3A4 abundance data source on *k*_cat_ (A) and intertissue *k*_cat_ ratio (B) in pooled human liver or intestinal microsomes. (Panel A) The bars represent mean *k*_cat_ ± standard deviation for 5 lots of pooled human liver and 5 lots of pooled human intestinal microsomes. (Panel B) All pairwise intestine: liver ratios for *k*_cat_ between 5 lots of pooled human liver and 5 lots of pooled human intestinal microsomes. The error bars represent the mean and standard deviation. Testosterone 6*β*-hydroxylation activity data were collated from vendors’ product information sheets for each microsomal lot (Supplemental Table 10). Literature average abundance represents the weighted mean of CYP3A4 abundance values collated from studies using similar proteomic methodology (liver: 3 studies^30^, ^31^, ^32^; intestine: 1 study^27^), same lab abundance represents CYP3A4 measured in individual liver and intestinal tissue within our laboratory using equivalent methods for all samples,^27^^,^^28^ same sample abundance represents the abundance measured in the specific lots of pooled human liver or intestinal microsomes for which the testosterone 6*β*-hydroxylation were measured (Supplemental Tables 11 and 12). CYP, cytochrome P450; *k*_cat_, turnover number (activity per unit of enzyme).

*k*_cat_ values for CYP3A4 were calculated for 5 lots of pooled HLM and 5 lots of pooled HIM using testosterone 6*β*-hydroxylation *V*_max_ data reported in vendors’ product data sheets (Supplemental Table 9). Data for other enzymes were typically not reported for the pooled HIM. To investigate the impact of the source of proteomics abundance data on estimation of *k*_cat_ values and corresponding ratios between different tissues, CYP3A4 *k*_cat_ for liver and intestine were calculated (eq. 2) using different sources of abundance data (see Section 2). The mean value of CYP3A4 *k*_cat_ for pooled HLM was 58% lower when using abundance data that had been measured in the same lot of microsomes that the activity data were generated in, compared with the *k*_cat_ value obtained using a literature average enzyme abundance (Fig. 1A). As expected, the interlot variability in liver *k*_cat_ was lower (CV = 7%) when using same sample abundance compared with literature average abundance (CV = 32%). In contrast, the impact of different sources of intestinal CYP3A4 abundance on the *k*_cat_ value for this enzyme was minimal (interlot CV = 51% for literature average abundance vs 49% for the same sample abundance). Of note, one of the pooled donor HIM lots (GYC) showed very low activity (163 pmol/min per mg protein) compared with the other HIM lots, despite having similar CYP3A4 abundance (18.6 pmol/mg protein) to the other donors (17.3–35.1 pmol/mg protein). Exclusion of this lot would reduce the interpool CVs for intestinal microsomes to approximately 22%.

The intestine:liver ratios of CYP3A4 *k*_cat_ calculated using all pairwise combinations of the 5 lots of pooled human liver and 5 of pooled HIM are shown in Fig. 1B. When using the literature average abundance or same lab abundance, the mean of the calculated intestine:liver CYP3A4 *k*_cat_ ratios were 0.47 and 0.59, which would imply that the CYP3A4 metabolic activity of each unit of enzyme is lower in the intestine compared with the liver. However, when using the same sample abundance, the mean of the calculated intestine:liver CYP3A4 *k*_cat_ ratios was 1.05, implying consistent metabolic activity of CYP3A4 independent of the tissue, in agreement with published literature (Supplemental Table 1). In addition to CYP3A4, measurements of enzyme activity and abundance to obtain *k*_cat_ and *k*_sp_ in pooled tissue microsomes were performed for other enzymes, revealing large interpool variability in *k*_cat_ and *k*_sp_ for some enzymes (Supplemental Fig. 1).

### 3.4 k_cat_ in matched liver and intestine from the same donors

**Fig. 2:**
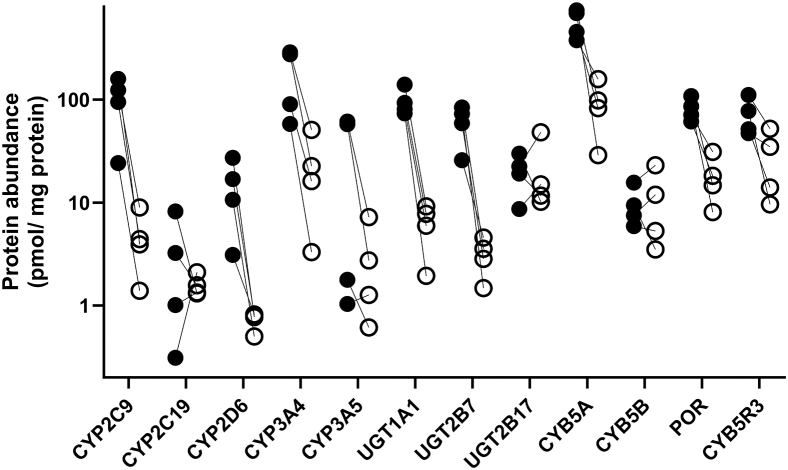
Quantification of selected drug-metabolizing enzymes and accessory proteins in paired human liver microsomes (closed symbols) and human intestinal microsomes (open symbols) from 4 donors. Abundances of cytochrome P450 (CYP) and uridine 5′-diphospho–glucuronosyltransferases (UGT) were quantified using targeted proteomics with QconCAT (MetCAT) standard, whereas accessory proteins cytochrome b_5_ subunit A (CYB5A) and subunit B (CYB5B), NADPH-cytochrome P450 reductase (POR), and NADH-cytochrome b_5_ reductase 3 (CYB5R3) were quantified using label-free global proteomics.

In vitro enzyme activity was measured in individual matched HLM and HIM from the same donors (D1–D4; Table 1) using specific probes of 7 metabolic enzymes investigated and assay conditions defined for pooled microsomal samples (Supplemental Tables 8 and 9). Donor D4 exhibited very low intestinal microsomal enzyme activity for all enzymes, with activity below the assay sensitivity limit for CYP2C9 (diclofenac depletion) and CYP2C19 ([*S*]-4-hydroxymephenytoin formation) (Supplemental Tables 8 and 9). Enzyme abundance data were generally higher in liver microsomes compared with the intestinal microsomes from the same donor for all the enzymes investigated. Among these, CYP2C9 and UGT2B7 showed the largest difference (up to 25-fold), whereas for other CYP enzymes, the fold difference between the liver and the intestine was between 10- and 20-fold (Fig. 2). In the case of CYP2C19 and UGT2B17, the abundances were comparable between the matched intestinal and liver microsomes. The abundances of accessory proteins were also quantified using label-free global proteomics. Cytochrome b_5_ showed similar abundance levels in the liver and the intestine, whereas NADPH-cytochrome P450 reductase and NADH-cytochrome b_5_ reductase 3 were 3-fold more abundant in the liver than in the intestine.

**Fig. 3:**
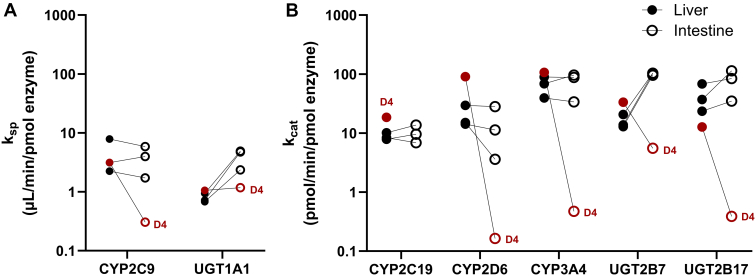
Individual donor enzyme activity after correction for enzyme abundance. Functional activity was measured for selected CYP and UGTs using substrate depletion (specificity constant [*k*_sp_]; *μ*L/min per pmol enzyme; Panel A) or metabolite formation approaches (turnover number [*k*_cat_]; pmol/min per pmol enzyme; Panel B) in human liver () and intestinal () microsomes prepared from 4 donors. The red circles represent donor D4 which had very low activity in intestinal microsomes (typically close to or below the limit of quantification). CYP, cytochrome P450; *k*_cat_, turnover number (activity per unit of enzyme); *k*_sp_, specificity constant; UGT, UDP-glucuronosyltransferase.

Correction of activity data by enzyme abundance revealed comparable enzyme activities when expressed per pmol enzyme between the liver and intestine for all CYP enzymes (CYP2C9, CYP2C19, CYP2D6, and CYP3A4). UGT1A1 and UGT2B7 were exceptions to this trend (Fig. 3, A and B). Donor D4 remained an outlier in the intestinal data set and was therefore excluded from subsequent calculations of intestine:liver *k*_cat_ ratios. Among the CYP enzymes investigated, the geometric mean of intestine:liver *k*_cat_ ratios ranged between 0.9 and 1.1; CYP2D6 was the exception with a geometric mean intestine:liver *k*_cat_ ratio of 0.56 (Supplemental Fig. 2). For intestinal CYP2D6, donor D3 had a lower *k*_cat_ compared with D1 and D2, as well as compared with hepatic CYP2D6 *k*_cat_ for D3. In addition, there was large variability in *V*_max_ between the enzymes (>400-fold), as the mean liver *V*_max_ was 27.6 and 11,289 pmol/min per mg protein for CYP2C19 and CYP3A4, respectively (Supplemental Tables 8 and 9). Conversely, the corresponding ratio between the lowest mean liver *k*_cat_ (11.2 pmol/min per pmol enzyme) and highest (76.1 pmol/min per pmol enzyme) was less than 7-fold.

**Fig. 4:**
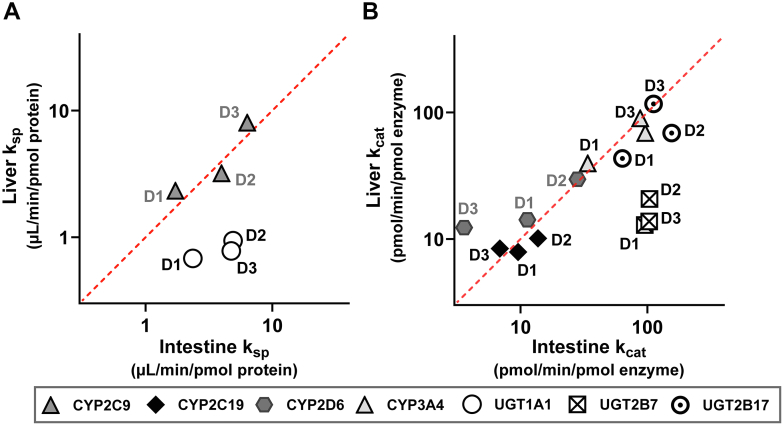
Comparison of intestine and liver specificity constant (*k*_sp_) (A) or turnover number (*k*_cat_) (B) for 4 cytochrome P450 (CYP) and 3 uridine 5′-diphospho–glucuronosyltransferase (UGT) enzymes in paired human microsomes from 3 donors. Enzyme activity data for each probe substrate are listed in Supplemental Tables 7 and 8. Enzyme abundances were quantified using targeted proteomics (Fig. 2). Data from donor D4 were excluded due to low activity in intestinal microsomes. The red dotted line represents the line of unity. *k*_cat_, turnover number (activity per unit of enzyme); *k*_sp_, specificity constant.

Interdonor variability in *k*_cat_ was observed, with a mean CV of 49% (range 5%–97%) after exclusion of data from HIM for donor D4. The donor-matched *k*_cat_ (and *k*_sp_) for intestine and liver were comparable, and distributed along the line of unity, suggesting interorgan correlation in *k*_cat_ values (Fig. 4, A and B). Even for UGT1A1 and UGT2B7, where ezetimibe *k*_sp_ and gemfibrozil glucuronide *k*_cat_ in HIM were 7- and 5-fold higher than in HLM, respectively (Fig. 3, A and B), the rank order of *k*_cat_ between donors was consistent between the organs (D2 > D3 > D1 in both cases) (Supplemental Fig. 3). Indeed, the *k*_cat_ (and *k*_sp_) values for donor D1 were typically below the mean for both liver and intestine (excluding D4), suggesting a common factor contributing to interdonor variability in *k*_cat_ and *k*_sp_. For other donors, such trends were not as consistent, for example, D2 had higher than average *k*_sp_ or *k*_cat_ for all enzymes in the intestine, but for the liver, D2 values were close to average (Fig. 4, A and B).

The contribution of CYP3A5 to 6-*β*-hydroxytestosterone formation was also evaluated. Calculated *k*_cat_ for 6-*β*-hydroxytestosterone formation was up to 50% lower when considering combined CYP3A4 and CYP3A5 abundance compared with considering only CYP3A4 abundance (Supplemental Table 14). However, these differences were comparable between the intestine and liver, resulting in a <10% difference in the corresponding intestine:liver *k*_cat_ ratio.

## 4 Discussion

PBPK models often assume that metabolic activity per unit of enzyme is comparable between the intestine and the liver, once specific tissue enzyme abundance is accounted for, based on the analysis performed for CYP3A4.^8^ From a purely biochemical perspective, this is a reasonable assumption. Variations may, in principle, arise if an enzyme is structurally different in 2 different tissues, for example, translation occurs using different start codons, differential folding, or enzymes are subjected to different post-translational modifications. LC-MS/MS proteomics does not normally provide insights into such variations. Furthermore, differential availability of cofactors and cosubstrates may also lead to differences in measured values of *k*_cat_. The current study aimed to measure the *k*_cat_ of multiple enzymes in paired human liver and intestinal microsomes from the same donors to reveal any cases where the assumption of tissue independent *k*_cat_ may be invalid.

### 4.1 Is metabolic activity (per unit enzyme) tissue independent?

Critical analysis of limited literature reports suggested comparable CYP2C9, CYP2C19, CYP2D6, and CYP3A4 metabolic activity per unit of enzyme between the intestine and liver, but the data for UGT1A1, UGT1A3, UGT1A6, and UGT2B7 remained uncertain (Supplemental Table 1). Our preliminary analysis of activity and abundance data measured in pooled human tissue microsomes illustrated the necessity to measure enzyme activity and expression in the same sample. Lower interlot variability in liver *k*_cat_ for CYP3A4 using the same sample abundance compared with using the literature average abundance was noted, resulting in more comparable intestine: liver *k*_cat_ ratios (Fig. 1). As such, many prior literature analyses, especially those focused on UGTs, are limited by the use of activity and proteomics data from different sources to calculate the enzymes’ *k*_cat_. Even when activity and abundance are measured in the same sample, the donors are not matched between pools from different tissues (Supplemental Fig. 1), which represents a key limitation of *k*_cat_ data from pooled tissue microsomes for intertissue comparisons. Furthermore, pooled HIM and HKM are generally comprised of a small number of donors (typically <15) in contrast to the liver (150–200 donors). As demonstrated for CYP2C9 and UGT1A1, these issues can result in highly variable calculated intertissue *k*_sp_ ratios that are dependent on the pair of pools selected for the analysis (Supplemental Fig. 1). To overcome the limitations of pooled microsomes, our analysis focused on paired human intestine and liver microsomes obtained from the same donor. Our results from paired human intestine and liver microsomes demonstrated that the *k*_cat_ or *k*_sp_ values for CYP2C9, CYP2C19, CYP2D6, and CYP3A4 were comparable between the 2 tissues (Fig. 4), in agreement with a critical analysis of sparse existing literature (Supplemental Table 1). These findings, albeit from only 3 donors, support the current common practice in drug development and PBPK modeling to extrapolate metabolic clearance data obtained in HLM to predict in vivo intestinal metabolism, once data are normalized for enzyme abundance differences between the liver and intestine (eg, CL_int_ expressed per pmol of relevant CYP enzyme). This pragmatic approach overcomes the current limitations in the quality and availability of HIM for in vitro drug metabolism studies. An alternative in vitro approach of using activity data from recombinantly expressed enzyme systems in conjunction with intersystem extrapolation factors that have been calibrated against HLM^6^^,^^33^ is also supported by our research findings.

In contrast, the results from our study with paired intestine and liver microsomes do not support the assumption that *k*_cat_ is tissue independent for UGTs. Intestinal *k*_sp_ and *k*_cat_ were 7- and 5-fold higher, respectively, than those in the liver for UGT1A1 and UGT2B7, with similar ratios observed for the 3 donors investigated (Supplemental Fig. 3). For UGT2B17, the intestine:liver *k*_cat_ ratios were variable between donors, ranging from 0.9- to 3.1-fold. The findings for UGT1A1 and UGT2B7 highlight the need for further investigation using matched samples to strengthen the evidence base. In contrast to the literature reports, standardized assay conditions were used here for each probe substrate to minimize the impact of experimental variability on *k*_cat_ between the tissues. The current study also accounted for albumin effect on UGT activity, but it is important to note that this effect is both enzyme and tissue-specific,^11^ likely due to differential release of free fatty acids during microsomal preparation from each tissue. Furthermore, enzyme kinetics (eg, Michaelis-Menten and allosteric) may differ between UGTs and tissues.^34^, ^35^, ^36^ In the current study, activity data were obtained at a single concentration due to limited sample availability for each donor, assuming Michaelis-Menten kinetics to derive *k*_cat_ and *k*_sp_. This assumption may be invalid for some UGT substrates that follow non–Michaelis-Menten kinetics.^35^ Finally, UGTs can show overlapping substrate specificity, which could confound interpretation of the activity data when the enzymes have differential expression patterns in each tissue; for example, glucuronidation of ezetimibe (probe for UGT1A1 in the current study) can be mediated by UGT1A1, UGT1A3, UGT2B7, and UGT2B15.^36^^,^^37^ To overcome this limitation, future experiments could measure *k*_cat_ by regressing *V*_max_ against abundance data from multiple donors. This approach, demonstrated for verapamil metabolite D-617 formation,^38^ revealed a nonzero intercept due to contributions from other enzymes (eg, CYP2C8 in the case of verapamil^39^). The geometric mean *k*_cat_ ratio for intestine:liver of 0.37 was lower than the slope ratio (1.14), likely reflecting a smaller CYP2C8 contribution in the intestine. Based on the current analyses, PBPK modelers are recommended to use a 7-fold uncertainty factor when using abundance-based scaling of UGT-mediated metabolism from HLM data to predict in vivo intestinal metabolism. This uncertainty factor is derived from the intertissue differences in UGT1A1 *k*_sp_ and UGT2B7 *k*_cat_ (Supplemental Fig. 3) and is expected to have the greatest impact on drugs undergoing extensive intestinal metabolism (ie, having a low fraction escaping first-pass effect in the gut [*F*_g_]). This sensitivity analysis using the Qgut model (Supplemental Fig. 4) showed that consideration of uncertainty in intertissue UGT *k*_cat_ had a significant impact on the predicted *F*_g_ for high intestinal extraction UGT substrates. Consideration of the intertissue differences in *k*_cat_ reduced the predicted *F*_g_ by up to 80%–90% for high extraction UGT substrates (such as raloxifene; Supplemental Table 15), whereas the effect was minimal for high *F*_g_ drugs. Sensitivity analysis in Supplemental Fig. 4 and in Supplemental Table 15 includes clinically used UGT drugs and their calculated percentage changes in predicted *F*_g_, depending on the assumption of the *k*_cat_ between the liver and intestine. However, as the UGT substrates in our data set mostly lack observed *F*_g_ data, further evaluation with UGT substrates with available observed human *F*_g_ data is needed.

These findings support the application of tissue-specific *k*_cat_ values or the inclusion of uncertainty factors in PBPK modeling and IVIVE for UGT substrates. No study has yet systematically evaluated *k*_cat_ in both liver and kidney for the same substrates in individual donor microsomes; although we were able to perform initial experiments in pooled HKM, acquiring individual donor kidney tissue samples of sufficient quality will be a challenge for future research.

PBPK models use a deterministic approach to propagate various biological sources of interindividual variability, generating virtual populations that are physiologically realistic and hence enable simulation-based analysis of pharmacokinetic variability.^40^ Robust abundance data are particularly valuable for difficult-to-study tissues where local drug disposition cannot easily be sampled or inferred from plasma measurements (eg, placenta and brain),^24^^,^^41^ or difficult-to-study population groups where clinical pharmacokinetics data are not easily attained (eg, pediatrics).^42^, ^43^, ^44^ These enzyme abundances, as visually reflected in pie charts presenting proportional enzyme abundances in specific tissues and populations,^45^ are often used to define distributions that describe interindividual variability in drug metabolism capacity. However, our research suggests that clinically relevant interindividual variability in drug metabolism may also come from differences in *k*_cat_.

A common assumption is that *k*_cat_ (or *k*_sp_) is independent of the biological system (ie, drug-specific). Conversely, our data suggest that there may be interindividual variability in *k*_cat_ (or *k*_sp_) for the same enzyme, with an average within-enzyme and within-tissue CVs of 49% (Fig. 3). PBPK models should take into account interenzyme correlations in abundance and activity.^46^ In our study, donor D1 had below average *k*_cat_ and *k*_sp_ for all 7 enzymes in both liver and intestine (Supplemental Fig. 2), indicating the possibility of interenzyme correlation in *k*_cat_, which needs to be evaluated further with a larger set of donors. Interindividual variability in *k*_cat_, calculated from literature data (only considering studies where activity and abundance were measured in the same sample, and with >10 donors), ranged 43%–218% based on geometric CV (Supplemental Table 1), in agreement with our findings. Such variability in *k*_cat_ is unlikely to be attributed to experimental variability, as technical precision for both activity and LC-MS abundance methods are considered to be high.^47^ Microsomal preparation can be affected by factors such as interoperator variability^4^ and tissue ischemia time. Enzyme activity is likely more sensitive to these factors than abundance measurements. Furthermore, intestinal microsomal preparation is likely to be more sensitive to these factors than the liver due to more complex protocols and the presence of endogenous intestinal proteolytic enzymes. Variability in *k*_cat_ can also arise due to true biological variability. The contribution of polymorphisms may apply for CYP2D6, CYP2C9, UGT2B7, and UGT2B17 but is not relevant for other enzymes.^48^ Although the limited number of donors precluded a robust statistical analysis, trends between *k*_cat_ and abundance of accessory proteins such as cytochrome b_5_ and NADPH-cytochrome P450 reductase in our individual donor microsomes (Fig. 2) were inconsistent. For example, hepatic CYP3A4 *k*_cat_ was the highest in donor D4 that also had the highest cytochrome b_5_, whereas no trend was noted for intestinal CYP3A4. Further, trends in *k*_cat_ were similar for CYPs and UGTs, suggesting that variability in *k*_cat_ may not be linked directly to the specific accessory proteins.

## 5 Conclusion

For the first time, the tissue dependence of enzyme activity (*k*_cat_ and *k*_sp_) has been evaluated across multiple CYP and UGT enzymes in paired donor intestinal and liver microsomes. Consistent with our preliminary data in pooled microsomes, *k*_sp_/*k*_cat_ for CYP2C9, CYP2C19, CYP2D6, and CYP3A4 were equivalent between the intestine and liver. Therefore, IVIVE-PBPK modeling of intestinal drug metabolism data via these enzymes by using HLM data normalized for enzyme abundance differences between the intestine and liver is a valid approach. Conversely, up to 7-fold higher *k*_sp_ or *k*_cat_ was observed for HIM data compared with HLM for UGT1A1 and UGT2B7. The reason for this discrepancy for UGTs is unclear and thus requires follow-up with additional substrates and a larger number of donors. In the meantime, it is recommended to perform sensitivity analyses when predicting intestinal first-pass metabolism using extrapolation from liver microsomal data or directly measure glucuronidation in intestinal microsomes.

## Conflict of interest

Amin Rostami-Hodjegan is an employee of Certara. All other authors declare no conflicts of interest.
