# Supplementary material for: Overlooked aspects of scaling enzyme activity through abundance across tissues and individuals: Insights from kcat measurements in matched liver and intestinal samples
Source: Drug Metab Dispos. 2025 Dec 29;54(4):100229. doi: 10.1016/j.dmd.2025.100229 (PMC13197946; doi:10.1016/j.dmd.2025.100229)
Supplement: Supplementary Material [file mmc1.docx]

**Supplementary materials**

**Overlooked aspects of scaling enzyme activity through abundance across tissues and individuals: Preliminary Insights from k_cat_ measurements in matched liver and intestinal samples**

Zubida M. Al-Majdoub, Jill Barber, Amin Rostami-Hodjegan, Aleksandra Galetin and Daniel Scotcher

Centre for Applied Pharmacokinetic Research, University of Manchester, Manchester, UK (Z.M.A.-M., J.B., A.R.-H., A.G., D.S.,); Certara Predictive Technologies, Sheffield, UK (A.R.-H.)

**Contents**

1. Supplemental Table 1. Summary of tissue ratios for activity per unit of enzyme (k_cat_) or specificity constant (k_cat_/ K_M_) obtained from published literature
2. Supplemental Table 2. In vitro substrate and microsomal protein concentrations, incubation time, and BSA concentrations (UGT substrates only) were selected to ensure first-order (substrate depletion) or zero-order (metabolite formation) conditions, and sufficient assay sensitivity
3. Section 1. Sample preparation for proteomics
4. Supplemental Table 3. Target proteins and their surrogate peptides in QconCAT standard (MetCAT) for cytochrome P450 (CYP) and uridine 5'-diphospho-glucuronosyltransferase (UGT) enzymes
5. Supplemental Table 4. Target proteins and their surrogate peptides used in label-free analysis
6. Supplemental Table 5. Literature analysis of CYP3A4 abundance in human liver microsomes
7. Supplemental Table 6. In vitro intrinsic clearance (CL_int_; µL/min/mg protein) in human liver, intestinal, and kidney microsomes from pooled donors measured using substrate depletion approach.
8. Supplemental Table 7. Metabolite formation rate at saturating substrate concentration (Vmax; pmol/min/mg protein) in human liver and intestinal microsomes from pooled donors.
9. Supplemental Table 8. In vitro intrinsic clearance (CL_int_; µL/min/mg protein) in human liver and intestinal microsomes from matched individual tissue donors measured using substrate depletion approach.
10. Supplemental Table 9. Metabolite formation rate at saturating substrate concentration (Vmax; pmol/min/mg protein) in human liver and intestinal microsomes from matched individual tissue donors.
11. Supplemental Table 10. 6-β hydroxy testosterone formation rate (Vmax; pmol/min/mg protein) in pooled donor human liver and intestinal microsomes reported in product information sheets provided by commercial vendor
12. Supplemental Table 11. Abundance of enzymes (pmol/mg of protein) in pooled donor human liver microsomes (HLM), quantified using targeted proteomics
13. Supplemental Table 12. Abundance of enzymes (pmol/mg of protein) in pooled donor human intestinal microsomes (HIM), quantified using targeted proteomics
14. Supplemental Table 13. Abundance of enzymes (pmol/mg of protein) in pooled donor human kidney microsomes (HKM), quantified using targeted proteomics
15. Supplemental Table 14. Comparison of 6-β hydroxy testosterone formation k_cat_ and intestine:liver k_cat_ ratio when considering abundance data either from CYP3A4 only, or the sum of CYP3A4 and CYP3A5, for calculating k_cat_
16. Supplemental Table 15. Effect of adjusted intestinal UGT activity on gut availability (Fg). Observed Fg for drugs primarily undergoing UGT-mediated metabolism, and potential impact of applying intestinal k_cat_ scaling factor (k_cat_ fold) on IVIVE-predictions of Fg
17. Supplemental Figure 1. Inter-tissue k_cat_ ratios for pooled human microsomes between intestine and liver or kidney and liver
18. Supplemental Figure 2. Intestine: liver k_cat_ ratios for three tissue donors
19. Supplemental Figure 3. Patterns of donor turnover number (k_cat_) or specificity constant (k_sp_) compared with mean for each enzyme in human liver or intestinal microsomes
20. Supplemental Figure 4. Sensitivity of predicted fraction escaping gut metabolism (Fg) from Qgut model from potential inaccurate assumption of turnover number (k_cat_) equivalence between human intestinal (HIM) and liver (HLM) microsomes
21. References

**Supplemental Table 1. Summary of tissue ratios for activity per unit of enzyme (k_cat_) or specificity constant (k_sp_) obtained from published literature ^a^.**

| **Enzyme (probe)** | **Organ vs liver** | **Tissue ratio (liver as reference ^c^)** | **Geometric CV ^b^ in k_cat_ or k_sp_** | **Biological samples** | **Limitations** | **Reference** |
| --- | --- | --- | --- | --- | --- | --- |
| CYP2C9 (diclofenac 4’-hydroxylation) | Intestine | 2.6 (k_cat_) **  1.5 (k_sp_) ** | Liver k_cat_: 63%  Liver k_sp_: 60%  Intestine k_cat_: 79%  Intestine k_sp_: 72% | Matched tissue from distal duodenum or proximal jejunum and liver (wedge biopsies)  N=12 patients who underwent gastrectomy or pancreatoduodenectomy | Proteomics by Western blot | Lapple et al, 2003 [1] |
| CYP2C9 (diclofenac 4’-hydroxylation) | Intestine | 1.0(k_cat_)  1.5 (k_sp_) | N/A | Activity measured in matched tissue biopsies from proximal jejunum and liver  N=20 patients with severe obesity undergoing Roux-en-Y gastric bypass surgery  In-house proteomics data used [2, 3] | Proteomics and activity data from different sources | Krogstad et al, 2004 [4] |
| CYP2C19 (S-mephenytoin 4'-hydroxylation) | Intestine | 0.9 (k_sp_) | Liver: 218%  Intestine: 97% | Matched tissue from distal duodenum or proximal jejunum and liver (wedge biopsies)  N=12 patients who underwent gastrectomy or pancreatoduodenectomy | Proteomics by Western blot | Lapple et al, 2003 [1] |
| CYP2D6 (bufuralol 1-hydroxylation) | Intestine | 2.1(k_cat_)  1.5 (k_sp_) | N/A | Activity measured in matched tissue biopsies from proximal jejunum and liver  N=20 patients with severe obesity undergoing Roux-en-Y gastric bypass surgery  In-house proteomics data used[2, 3] | Proteomics and activity data from different sources | Krogstad et al, 2004 [4] |
| CYP3A4 (verapamil 🡪 D-617)  (verapamil 🡪 norverapamil) | Intestine | 1.2 (k_cat_) *  1.1 (k_cat_) * | Liver: 161%  Intestine: 43% | Tissue from distal duodenum or proximal jejunum and liver (wedge biopsies)  N=15 patients who underwent gastrectomy or pancreatoduodenectomy | Proteomics by Western blot | Yang et al, 2004[5], with data reported by von Richter et al, 2002[6] |
| CYP3A4  (22 substrates) | Intestine | 1.3 (k_sp_) | N/A | Activity measured in N=3 liver microsomal pools (22 – 50 donors) and N=1 intestinal microsomal pool (10 donors)  Abundance from literature data |  | Gertz et al, 2010 [7] |
| CYP3A4 (midazolam 1-hydroxylation) | Intestine | - 1. (k_cat_)   1.7 (k_sp_) | N/A | Activity measured in matched tissue biopsies from proximal jejunum and liver  N=20 patients with severe obesity undergoing Roux-en-Y gastric bypass surgery  In-house proteomics data used [2, 3] | Proteomics and activity data from different sources | Krogstad et al, 2004 [4] |
| UGT1A1 (ezetimibe) | Intestine | 15.1 (k_cat_)  4.9 (k_sp_) | N/A | Literature analysis [8] | Ezetimibe also substrate of UGT1A3, and UGT2B15 in liver, and UGT2B17 in intestine [9]  Proteomics and activity data from different sources | Ahmed et al, 2022 [10] |
| UGT1A3 (telmisartan) | Intestine | 6.37 (k_sp_) | N/A | Literature analysis | Proteomics and activity data from different sources | Ahmed et al, 2022 [10] |
| UGT1A6 (deferiprone) | Intestine | 3.5 (k_cat_)  6.4 (k_sp_) | N/A | Literature analysis [8, 11] | Kinetics differed between liver (Michaelis-Menten) and intestine/ kidney (Hill equation)  Proteomics and activity data from different sources | Ahmed et al, 2022 [10] |
| UGT2B7 (naloxone) | Intestine | 39.9 (k_sp_) | N/A | Literature analysis | Intestine:liver ratio highly variable (>1-order magnitude)  Proteomics and activity data from different sources | Ahmed et al, 2022 [10] |
| UGT1A1 (ezetimibe) | Kidney | 28.1 (k_sp_) | N/A | Literature analysis | Ezetimibe also substrate of UGT1A3, and UGT2B15 in liver [9]  Proteomics and activity data from different sources [12] | Ahmed et al, 2022 [10] |
| UGT1A3 (telmisartan) | Kidney | 19.4 (k_sp_) | N/A | Literature analysis | Proteomics and activity data from different sources | Ahmed et al, 2022 [10] |
| UGT1A6 (deferiprone) | Kidney | 65.9 (k_cat_) | N/A | Literature analysis [11, 13, 14] | Proteomics and activity data from different sources [12] | Ahmed et al, 2022 [10] |
| UGT1A9 (mycophenolic acid)  (propofol) | Kidney | 11.9 (k_cat_)  63.7 (k_sp_)  39.4 (k_cat_)  53.3 (k_sp_) | N/A | Literature analysis [13, 14] | Proteomics and activity data from different sources | Ahmed et al, 2022 [10] |
| UGT2B7 (naloxone)  (zidovudine) | Kidney | 239 (k_cat_)  104 (k_sp_)  1.4 (k_cat_) | N/A | Literature analysis [13, 14] | Proteomics and activity data from different sources | Ahmed et al, 2022 [10] |

^a^ Criteria for inclusion: In vitro activity measured for human drug metabolising enzymes using selective probe substrates in liver and intestine or kidney microsomes in the same study and with consistent methodology

^b^ Geometric CV = sqrt(exp(σ^2^) – 1), where σ is the arithmetic standard deviation of log(X), only reported where abundance and activity were measured in the same sample

^c^ Tissue ratio represents intestine:liver or kidney:liver ratios, where ratio of 1 means that the k_cat_ or specificity constant is the same between the tissues.

* k_cat_ obtained from slope of linear regression between activity and abundance

** k_cat_ obtained from correction of activity for abundance at sample level

**Supplemental Table 2. In vitro experimental conditions for functional activity assays in human liver (HLM), kidney (HKM) and intestinal (HIM) microsomes**

| **Substrate**  **(enzyme)** | **Diclofenac**  **(CYP2C9)** | **S-mephenytoin**  **(CYP2C19)** | **Dextromethorphan**  **(CYP2D6)** | **Testosterone**  **(CYP3A4)** | **Ezetimibe**  **(UGT1A1)** | **Deferiprone (UGT1A6)** | **Propofol**  **(UGT1A9)** | **Gemfibrozil**  **(UGT2B7)** | **Testosterone (UGT2B17)** |
| --- | --- | --- | --- | --- | --- | --- | --- | --- | --- |
| **Assay format** | Depletion^a^ | (S)-4-Hydroxy mephenytoin formation | Dextrorphan formation | 6-β hydroxy testosterone formation | Depletion^a^ | Depletion^a^ | Depletion^a^ | Depletion^a^ and gemfibrozil glucuronide formation^b^ | Testosterone glucuronide formation |
| **Substrate initial concentration (µM) [K_m_]** | HIM: 0.1 µM  HLM: 1 µM  [5 – 50 µM] [15-17] | 500  [106 µM (HLM), 30 µM (HIM)][18] | 500  [3 µM, 156 µM ^c^][19] | 250  [55 µM (HLM), 56 µM (HIM)][18] | 1  [21 µM][20] | 1  [7.6 – 10.0 µM in HLM; Non-MM kinetics in HIM and HKM][11] | 5  [205 µM][21] | Depletion: 1  Formation: 125 ^d^  [2.5, 121 µM ^c^][22] | 250  [4 µM, 14 µM ^e^][23] |
| **Protein concentration (mg/mL)** | 0.5 | 1 | 0.5 | 0.5 | 0.25 | 0.3 | 0.5 | 1 | 0.25 |
| **Incubation time (min)** | 45 | 60 | 60 | 10 | 30 | 60 | 40 | 60 | 30 |
| **Cofactor** | 1mM NADPH | | | | 5 mM glucuronic acid | | | | |
| **Bovine serum albumin concentration** | N/A | N/A | N/A | N/A | 2% | N/A | 2% | 1% | .2% |

Assays were run with triplicate wells, except for individual intestinal microsomes for donor D3 which were run in duplicate due to sample availability

^a^ Limit of sensitivity was set at minimum 10% depletion by final timepoint, considering the high reproducibility of the depletion assay (relative standard error of slope were typically <10%)

^b^ Due to low sensitivity of depletion assay using pooled microsomes, gemfibrozil glucuronide formation was monitored in the experiments with individual donor microsomes.

^c^ Values represent reported high affinity, low capacity and low affinity, high capacity sites.

^d^ Substrate concentration limited by solubility considerations

^e^ Values reported for HLM donors with high and medium expression of UGT2B17

**Section 1**. Sample preparation for proteomics

Sample mixtures were solubilized by incubation with sodium deoxycholate (10% w/v final volume), 1,4-dithiothreitol (DDT) was added at a final concentration of 100 mM, and the protein mixture was incubated at room temperature for 10 min. Reduction of protein disulfide bonds was carried out by incubation at 56°C for 40 min. Amicon Ultra 0.5 mL centrifugal filters with 10-kDa molecular mass cutoff (Millipore, Nottingham, UK) were conditioned by briefly centrifuging 400 mL of 0.1 M Tris of pH 8.5 at 14000 g at room temperature. The protein samples were then transferred to the conditioned filter units, and this was followed by centrifugation at 14000 g at room temperature for 30 min. Alkylation of reduced cysteine was performed by incubation with 100 mL of 50 mM iodoacetamide in the dark for 30 min at room temperature. After alkylation, deoxycholate removal was performed by buffer exchange using two successive washes with 8 M urea in 100 mM Tris-HCl, pH 8.5, 200 mL each. To reduce urea concentration, additional washes (3 × 200 mL) were performed using 1 M urea in 50 mM ammonium bicarbonate pH 8.5. For each wash, 200 mL buffer was added to the filter without mixing and centrifuged at 14000 g at room temperature for 20 min, leaving a volume of approximately 20 µL in the filter. The filtrate containing small molecules, such as detergent, was discarded. Protein digestion was achieved using Lys C twice (Lys C:protein ratio 1:50, 2 h each, at 30°C), and then trypsin digestion was carried out (trypsin:protein ratio 1:25) for 14 h at 37°C as well as another equivalent treatment of an extra 6 h incubation. Peptides were recovered from the filter by centrifugation (14000 g, 20 min); a second collection was achieved by adding 0.5 M sodium chloride (100 mL) to the filter and centrifuged at 14000 g for another 20 min. The collected peptides were lyophilized to dryness using a vacuum concentrator at 30°C with vacuum in aqueous mode; the time required was in the range 1–3 h and was sample-dependent. Lyophilized peptides were reconstituted in 20% (v/v) acetonitrile in water, acidified with 2% (v/v) trifluoroacetic acid, and then desalted using C18 spin columns according to the manufacturer’s instructions. The peptides were lyophilized and stored at 80°C until mass spectrometric analysis.

**Supplemental Table 3. Target proteins and their surrogate peptides in QconCAT standard (MetCAT) for cytochrome P450 (CYP) and uridine 5'-diphospho-glucuronosyltransferase (UGT) enzymes**

| **Target protein** | **Surrogate Peptides** |
| --- | --- |
| **CYP2C9** | GIFPLAER^a^, LPPGPTPLPVIGNILQIGIK |
| **CYP2C19** | GHFPLAER^a^ |
| **CYP2D6** | AFLTQLDELLTEHR ^a^, DIEVQGFR^a^ |
| **CYP3A4** | EVTNFLR^a^, LSLGGLLQPEK^a^ |
| **CYP3A5** | DTINFLSK^a^, YWTEPEEFRPER |
| **UGT1A1** | DGAFYTLK^a^, TYPVPFQR |
| **UGT1A6** | VSVWLLR^a^, SFLTAPQTEYR^a^ |
| **UGT1A9** | AFAHAQWK^a^, ESSFDAVFLDPFDNCGLIVAK, ESSFDAVFLDPFDNCGLIVAK |
| **UGT2B7** | ADVWLIR, TILDELIQR^a^ |
| **UGT2B15** | SVINDPVYK^a^, WIYGVSK^a^ |

^a^Peptides used for the quantification of the targets in the targeted analysis

**Supplemental Table 4. Target proteins and their surrogate peptides used in label-free analysis**

| **Target proteins** | **Surrogate peptides** |
| --- | --- |
| CYB5A | FLEEHPGGEEVLR, YYTLEEIQK, TFIIGELHPDDRPK |
| CYB5B | FLNEHPGGEEVLLEQAGVDASESFEDVGHSSDAR, GQEVETSVTYYR, QYYIGDIHPSDLKPESGSK |
| CYB5R3 | DILLRPELEELR, IDGNLVVRPYTPISSDDDK, DPDDHTVCHLLFANQTEK |
| POR | EVGETLLYYGCR, IRYESGDHVAVYPANDSALVNQLGK, NIIVFYGSQTGTAEEFANR |

Cytochrome b5 type A (CYB5A); Cytochrome b5 type B (CYB5B); NADH-cytochrome b5 reductase 3 (CYB5R3); NADPH- cytochrome P450 reductase (POR)

**Supplemental Table 5 Literature analysis of CYP3A4 abundance in human liver microsomes ^a^**

| **Study #** | **Source of microsomes** | **Number of donors** | **CYP3A4 abundance (mean ± SD)**  **[pmol/mg protein]** |
| --- | --- | --- | --- |
| **1** | Commercial | 17 | 60.4 ± 75.0 |
| **2** | Commercial | 24 | 68.1± 52.3 |
| **3** | In-house | 100 | 49.3 ^b^ |
| **Weighted mean** | **-** | **141** | **53.8** |

^a^ Inclusion criteria were use of targeted proteomics in human liver microsomes prepared from donors without specific liver diseases (e.g., [24-26]. Data reported in homogenates or S9 fractions, or generated using global proteomics were not included [27, 28]. ^b^ Median value reported by study 3 was used in calculation of the weighted mean.

**Supplemental Table 6. In vitro intrinsic clearance (CL_int_; µL/min/mg protein) in human liver, intestinal, and kidney microsomes from pooled donors measured using substrate depletion approach.**

| Pool | Tissue | Diclofenac  (CYP2C9) | Ezetimibe  (UGT1A1) | Deferiprone (UGT1A6) | Propofol  (UGT1A9) | Gemfibrozil  (UGT2B7) |
| --- | --- | --- | --- | --- | --- | --- |
| BRE | **Liver** | 245.8 | 66.4 | 13.5 | - | - |
| 36170 | **Liver** | 251.5 | 1255.0 | - | 59.2^a^ | 77.8 |
| 38290 | **Liver** | 187.0 | 1045.0 | - | 62.4^a^ | 74.3 |
| GYC | **Intestine** | 4.4 ^b^ | 3.2 | ND | - | - |
| 1110396 | **Intestine** | 8.9 | 34.4 | - | 24.1^a^ | 4.0 |
| 1610314 | **Intestine** | 8.3 | 31.7 | - | 25.9^a^ | 1.3 |
| 1710160 | **Kidney** | 6.2 | 14.6 | - | 186.3 | 20.0 |
| 1710120 | **Kidney** | 1.3 | 18.6 | - | 183.3 | 16.4 |

^a^ Similar depletion observed in no-cofactor control

^b^ relative standard error of slope >40%, indicating possible poor precision of measurement

ND – Insufficient depletion during assay to obtain CL_int_

**Supplemental Table 7. Metabolite formation rate at saturating substrate concentration (Vmax; pmol/min/mg protein) in human liver and intestinal microsomes from pooled donors.**

| **Pool** | **Tissue** | **(S)-4-Hydroxy mephenytoin**  **(CYP2C19)** | **Dextrorphan**  **(CYP2D6)** | **6-β hydroxy testosterone**  **(CYP3A4)** | **Gemfibrozil glucuronide**  **(UGT2B7)** | **Testosterone glucuronide**  **(UGT2B17)** |
| --- | --- | --- | --- | --- | --- | --- |
| **BRE** | **Liver** | 52.2 | 372.9 | 7770.1 | 992.1 | 299.5 |
| **GYC** | **Intestine** | 0.6 | 1.2 | 212.6 | 61.3 | 369.0 |

**Supplemental Table 8. In vitro intrinsic clearance (CL_int_; µL/min/mg protein) in human liver and intestinal microsomes from matched individual tissue donors measured using substrate depletion approach.**

|  | **Diclofenac**  **(CYP2C9)** | | **Ezetimibe**  **(UGT1A1)** | |
| --- | --- | --- | --- | --- |
| Donor | **Liver** | **Intestine** | **Liver** | **Intestine** |
| D1 | 277.5 | 15.4 | 95.1 | 21.6 |
| D2 | 497.6 | 15.6 | 88.0 | 38.2 |
| D3 | 190.5 | 8.1 ^b^ | 52.9 | 9.2 ^b^ |
| D4 ^a^ | 299.7 | 1.3 ^c^ | 85.3 | 7.0 ^c^ |

^a^ Donor D4 was excluded from subsequent analysis due to overall low activity in the intestinal microsomes

^b^ RSE of slope >40%, indicating possible poor precision of measurement

^c^ <10% depletion by last timepoint, and relative standard error (RSE) > 100%, suggesting poor precision of measurement

**Supplemental Table 9. Metabolite formation rate at saturating substrate concentration (Vmax; pmol/min/mg protein) in human liver and intestinal microsomes from matched individual tissue donors.**

|  | **(S)-4-Hydroxy mephenytoin**  **(CYP2C19)** | | **Dextrorphan**  **(CYP2D6)** | | **6-β hydroxy testosterone**  **(CYP3A4)** | | **Gemfibrozil glucuronide**  **(UGT2B7)** | | **Testosterone glucuronide**  **(UGT2B17)** | |
| --- | --- | --- | --- | --- | --- | --- | --- | --- | --- | --- |
| **Donor** | **Liver** | **Intestine** | **Liver** | **Intestine** | **Liver** | **Intestine** | **Liver** | **Intestine** | **Liver** | **Intestine** |
| **D1** | 25.6 | 15.0 | 386.2 | 8.7 | 11399.2 | 1722.1 | 1084.5 | 339.6 | 530.0 | 1683.7 |
| **D2** | 10.2 | 18.4 | 315.1 | 14.0 | 18928.3 | 2169.4 | 1219.2 | 295.3 | 1110.5 | 1157.8 |
| **D3** | 68.8 | 9.0 | 255.8 | 2.9 | 5187.8 | 289.4 | 1001.9 | 151.7 | 1305.7 | 978.9 |
| **D4 ^a^** | 5.7 | ND | 279.8 | 0.1 ^b^ | 9641.2 | 7.7 ^b^ | 861.7 | 25.4 | 109.7 | 5.8 |

^a^ Donor D4 was excluded from subsequent analysis due to overall low activity in the intestinal microsomes

^b^ Relative standard error (RSE) for Vmax >100% due to slow formation of metabolite indicating poor precision of measurement (RSE for all other values <20%)

ND – not determined because metabolite concentration was below lower limit of quantification

**Supplemental Table 10. 6-β hydroxy testosterone formation rate (Vmax; pmol/min/mg protein) in pooled donor human liver and intestinal microsomes reported in product information sheets provided by commercial vendor**

| Lot number | Tissue | 6-β hydroxy testosterone formation rate  (pmol/min/mg protein) |
| --- | --- | --- |
| 36170 | Liver | 5900 |
| 38290 | Liver | 4600 |
| 88114 | Liver | 5700 |
| 38289 | Liver | 5300 |
| BRE | Liver | 2153 |
| 1110396 | Intestine | 1260 |
| 1610314 | Intestine | 951 |
| 610108 | Intestine | 1510 |
| 0710352 | Intestine | 1060 |
| GYC | Intestine | 163 |

**Supplemental Table 11. Abundance of enzymes (pmol/mg of protein) in pooled donor human liver microsomes (HLM), quantified using targeted proteomics**

| **Enzyme** | **Lot number** | | | | |
| --- | --- | --- | --- | --- | --- |
|  | **38289** | **88114** | **38290** | **36170** | **BRE** |
| **CYP2C9** | NA | NA | 155.3 | 141.4 | 56.7 |
| **CYP2C19** | NA | NA | NA | NA | 3.0 |
| **CYP2D6** | NA | NA | NA | NA | 7.8 |
| **CYP3A4** | 129.0 | 168.4 | 130.6 | 158.7 | 57.0 |
| **UGT1A1** | NA | NA | **ND** | 29.1 | 37.7 |
| **UGT1A6** | NA | NA | NA | NA | **ND** |
| **UGT1A9** | NA | NA | 131.0 | 97.3 | 15.7 |
| **UGT2B7** | NA | NA | 194.3 | 123.7 | 23.4 |
| **UGT2B17** | NA | NA | NA | NA | 8.9 |

NA; not analysed, **ND**; not detected

**Supplemental Table 12. Abundance of enzymes (pmol/mg of protein) in pooled donor human intestinal microsomes (HIM), quantified using targeted proteomics**

| **Enzyme** | **Lot number** | | | | |
| --- | --- | --- | --- | --- | --- |
|  | **1110396** | **1610314** | **610108** | **0710352** | **GYC** |
| **CYP2C9** | 4.8 | 2.8 | NA | NA | 5.2 |
| **CYP2C19** | NA | NA | NA | NA | 1.5 |
| **CYP2D6** | NA | NA | NA | NA | 0.8 |
| **CYP3A4** | 28.9 | 25.4 | 35.1 | 17.3 | 18.6 |
| **UGT1A1** | 9.7 | 10.3 | NA | NA | 8.3 |
| **UGT1A6** | NA | NA | NA | NA | **ND** |
| **UGT1A9** | **ND** | **ND** | NA | NA | 0.2 |
| **UGT2B7** | 7.5 | 4.6 | NA | NA | 3.2 |
| **UGT2B17** | NA | NA | NA | NA | 11.7 |

NA; not analysed, **ND**; not detected

**Supplemental Table 13. Abundance of enzymes (pmol/mg of protein) in pooled donor human kidney microsomes (HKM), quantified using targeted proteomics**

| **Enzyme** | **Lot number** | |
| --- | --- | --- |
|  | **1410120** | **1710160** |
| **CYP2C9** | 2.1 | **ND** |
| **CYP2C19** | NA | NA |
| **CYP2D6** | NA | NA |
| **CYP3A4** | NA | NA |
| **UGT1A1** | **ND** | **ND** |
| **UGT1A6** | NA | NA |
| **UGT1A9** | 52.5 | 239.0 |
| **UGT2B7** | 90.3 | 61.6 |
| **UGT2B17** | NA | NA |

NA; not analysed, **ND**; not detected

**Supplemental Figure 1**. **Inter-tissue k_cat_ ratios for pooled human microsomes between intestine and liver or kidney and liver.** Activity of CYP2C9, UGT1A1, UGT1A9, and UGT2B7 were measured using assays for diclofenac depletion, ezetimibe depletion, propofol depletion, and gemfibrozil glucuronide formation, respectively. Bars and error bars represent median and range for all pairwise k_cat_ or k_sp_ ratios between multiple lots of pooled human liver and multiple lots of pooled intestine or kidney intestinal microsomes. Activity and abundance data were measured for each pool.

**Supplemental Figure 2. Intestine: liver k_cat_ ratios for three tissue donors**. k_cat_ values for several CYP and UGT enzymes calculated using Vmax (pmol/min/mg protein) except CYP2C9 and UGT1A1, the k_sp_ was calculated using CL_int_ (µl/min/mg protein) values. Donor D4 was excluded.

 **Supplemental Figure 3. Patterns of donor turnover number (k_cat_) or specificity constant (k_sp_) compared with mean for each enzyme in human liver or intestinal microsomes.** Differences for each donor are expressed as % difference compared with the mean of n=4 (liver) or n=3 (intestine; D4 excluded) donors.

**
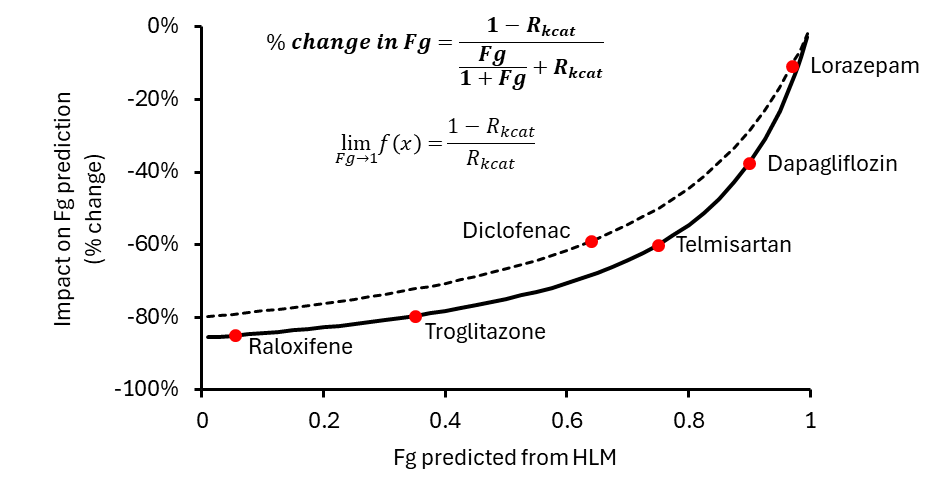
**

**Supplemental Figure 4. Sensitivity of predicted fraction escaping gut metabolism (Fg) from Qgut model from potential inaccurate assumption of** **turnover number (k_cat_) equivalence between human intestinal (HIM) and liver (HLM) microsomes.** A series of hypothetical drugs were generated with range of predicted Fg using the Qgut model and assuming k_cat_ is same between HIM and HLM. For each drug, the % change in Fg represents the difference in Fg between the assumption that k_cat_ is same in HIM and HLM (i.e., as denominator/ reference value), and assumption that k_cat_ in HIM is up to 7-fold (solid line) or 5-fold higher (dashed line) than in HLM, as suggested by our experimental data for UGT1A or UGT2B families, respectively. Example UGT substrate drugs (raloxifene, troglitazone, telmisartan, diclofenac, dapagliflozin, lorazepam) are indicated by the coloured markers overlaid on the curve, corresponding to their clinical Fg values (Table S15). For UGTs with a 7-fold higher k_cat_ in intestine, the model predicts up to 80-90% reduction in Fg for drugs with extensive gut metabolism.

Derivation of equation S9 used for Supplemental Figure 4:

Starting with the Qgut model[29]

| $Fg= \frac{Q_{gut}}{Q_{gut}+{fu}_{G}\cdot{CL}_{int,u,G}}$ | *Eq. S1* |
| --- | --- |
| $Q_{gut}= \frac{Q_{villi}\cdot{CL}_{perm}}{Q_{villi}+{CL}_{perm}}$ | *Eq. S2* |

Where Fg, Qgut, fu_G_, CL_int,u,G_, and CL_perm_ are the fraction escaping gut metabolism, “hybrid” flow term, fraction unbound in enterocytes, unbound intrinsic clearance in gut, and permeability clearance across the enterocyte plasma membrane, respectively.

To compare the difference in Fg between two scenarios where only the CL_int,u,G_ is changing between them “% change in Fg” can be defined based on a reference Fg (“Fg_1_”), and a new Fg value (“Fg_2_”). In addition, the k_cat_ ratio (“R_kcat_”) can be defined as representing the fold-difference between the corresponding CL_int,u,G_ (representing potential misprediction of in vitro-in vivo extrapolation from human liver microsomes).

| $R_{kcat}= \frac{{CL}_{int,u,G,2}}{{CL}_{int,u,G,1}}$ | *Eq. S3* |
| --- | --- |
| $\% change in Fg=\frac{{Fg}_{2}-{Fg}_{1}}{{Fg}_{1}}= \frac{\frac{Q_{gut}}{Q_{gut}+{fu}_{G}\cdot R_{kcat}\cdot{CL}_{int,u,G,1}}- \frac{Q_{gut}}{Q_{gut}+{fu}_{G}\cdot{CL}_{int,u,G,1}}}{\frac{Q_{gut}}{Q_{gut}+{fu}_{G}\cdot{CL}_{int,u,G,1}}}$ | *Eq. S4* |

Rearrange and simplify

| $\% change in Fg\cdot\frac{1}{Q_{gut}+{fu}_{G}\cdot{CL}_{int,u,G,1}}= \frac{1}{Q_{gut}+{fu}_{G}\cdot R_{kcat}\cdot{CL}_{int,u,G,1}}- \frac{1}{Q_{gut}+{fu}_{G}\cdot{CL}_{int,u,G,1}}$ | *Eq. S5* |
| --- | --- |
| $\% change in Fg= \frac{Q_{gut}+{fu}_{G}\cdot{CL}_{int,u,G,1}}{Q_{gut}+{fu}_{G}\cdot R_{kcat}\cdot{CL}_{int,u,G,1}}- 1$ | *Eq. S6* |
| $\% change in Fg= \frac{1-R_{kcat}}{\frac{Q_{gut}}{{fu}_{G}\cdot{CL}_{int,u,G,1}}+R_{kcat}}$ | *Eq. S7* |

Rearrange Q_gut_ equation (expressed as $\frac{Q_{\mathrm{gut}}}{\mathrm{fu}_{G}\cdot\mathrm{CL}_{int,u,G,1}}$)

| $Fg= \frac{Q_{gut}}{Q_{gut}+{fu}_{G}\cdot{CL}_{int,u,G}} \boldsymbol{\to} \frac{Q_{gut}}{{fu}_{G}\cdot{CL}_{int,u,G,1}}= \frac{Fg}{1-Fg}$ | *Eq. S8* |
| --- | --- |

Substitute Eq. S8 into Eq. S7

| $\boldsymbol{\% change in Fg}\boldsymbol{=} \frac{\boldsymbol{1-}\boldsymbol{R}_{\boldsymbol{kcat}}}{\frac{\boldsymbol{Fg}}{\boldsymbol{1-Fg}}\boldsymbol{+}\boldsymbol{R}_{\boldsymbol{kcat}}}$ | ***Eq. S9*** |
| --- | --- |

**Supplemental Table 14. Comparison of 6-β hydroxy testosterone formation k_cat_ and intestine:liver k_cat_ ratio when considering abundance data either from CYP3A4 only, or the sum of CYP3A4 and CYP3A5, for calculating k_cat_**

| Donor | k_cat_ (pmol/min/pmol enzyme) | | | | Intestine:Liver k_cat_ ratio | | |
| --- | --- | --- | --- | --- | --- | --- | --- |
|  | Liver | | Intestine | | CYP3A4 only | CYP3A4 + CYP3A5 | % difference |
|  | CYP3A4 only | CYP3A4 + CYP3A5 | CYP3A4 only | CYP3A4 + CYP3A5 |  |  |  |
| **D1** | 39.5 | 32.6 | 33.8 | 29.6 | 0.86 | 0.91 | 6% |
| **D2** | 68.6 | 68.2 | 96.1 | 93.6 | 1.40 | 1.37 | -2% |
| **D3** | 89.5 | 44.8 | 87.7 | 47.9 | 0.98 | 1.07 | 9% |
| **D4** | 106.8 | 105.6 | 0.5 | 0.4 | ND | ND | ND |

ND – k_cat_ ratio not calculated for D4 due to very low activity in intestinal microsomes

**Supplemental Table 15. Effect of adjusted intestinal UGT activity on gut availability (Fg)**. **Observed Fg for drugs primarily undergoing UGT-mediated metabolism, and potential impact of applying intestinal k_cat_ scaling factor (k_cat_ fold) on IVIVE-predictions of Fg.**

| **Drug** | **Fg observed** | **Intestinal UGT involvement^a^** | **k_cat_ fold^b^** | **Impact of k_cat_ assumption on Fg prediction from HLM^c^** | **Reference** |
| --- | --- | --- | --- | --- | --- |
| Raloxifene | 0.054 | UGT1A1 | 7x | -85% | Mizuma et al, 2009 [30] |
| Troglitazone | 0.35 | UGT1A1 | 7x | -80% | Cubitt et al, 2009 [31] |
| Diclofenac | 0.64 | UGT2B7 | 5X | -59% | Varma et al, 2010 [32] |
| Telmisartan | 0.75 | UGT1A3 | 7x | -60% | Varma et al, 2010 [32] |
| Dapagliflozin | 0.90 | UGT1A subfamily | 7x | -38% | Boulton et al, 2013 [33] |
| Lorazepam | 0.97 | UGT2B7 | 5x | -11% | Varma et al, 2010 [32] |
|  |  |  |  |  |  |

^a^ Dominant UGT enzyme(s) implicated for each drug.

^b^ Fold-increase in intestinal versus hepatic k_cat_ based on experimental measurements from matched human donor microsomes. Values of 7× applied to UGT1A subfamily enzymes; 5× applied to UGT2B7.

^c^ Equation S9; negative values indicate a decrease in predicted Fg using human liver microsomes after applying the k_cat_ scaling factor compared to predicted Fg without the scaling factor.

**References**

[1] Lapple F, von Richter O, Fromm MF *et al.* Differential expression and function of CYP2C isoforms in human intestine and liver. Pharmacogenetics 2003; 13:565-575.

[2] Couto N, Al-Majdoub ZM, Gibson S *et al.* Quantitative Proteomics of Clinically Relevant Drug-Metabolizing Enzymes and Drug Transporters and Their Intercorrelations in the Human Small Intestine. Drug Metab Dispos 2020; 48:245-254.

[3] Achour B, Barber J, Rostami-Hodjegan A. Expression of hepatic drug-metabolizing cytochrome p450 enzymes and their intercorrelations: a meta-analysis. Drug Metab Dispos 2014; 42:1349-1356.

[4] Krogstad V, Peric A, Robertsen I *et al.* A Comparative Analysis of Cytochrome P450 Activities in Paired Liver and Small Intestinal Samples from Patients with Obesity. Drug Metab Dispos 2020; 48:8-17.

[5] Yang J, Tucker GT, Rostami-Hodjegan A. Cytochrome P450 3A expression and activity in the human small intestine. Clin Pharmacol Ther 2004; 76:391.

[6] von Richter O, Burk O, Fromm MF *et al.* Cytochrome P450 3A4 and P-glycoprotein expression in human small intestinal enterocytes and hepatocytes: a comparative analysis in paired tissue specimens. Clin Pharmacol Ther 2004; 75:172-183.

[7] Gertz M, Harrison A, Houston JB, Galetin A. Prediction of human intestinal first-pass metabolism of 25 CYP3A substrates from in vitro clearance and permeability data. Drug Metab Dispos 2010; 38:1147-1158.

[8] Margaillan G, Rouleau M, Klein K *et al.* Multiplexed Targeted Quantitative Proteomics Predicts Hepatic Glucuronidation Potential. Drug Metab Dispos 2015; 43:1331-1335.

[9] Ghosal A, Yuan Y, Hapangama N *et al.* Identification of human UDP-glucuronosyltransferase enzyme(s) responsible for the glucuronidation of 3-hydroxydesloratadine. Biopharm Drug Dispos 2004; 25:243-252.

[10] Ahmed AN, Rostami-Hodjegan A, Barber J, Al-Majdoub ZM. Examining Physiologically Based Pharmacokinetic Model Assumptions for Cross-Tissue Similarity of Activity per Unit of Enzyme: The Case Example of Uridine 5'-Diphosphate Glucuronosyltransferase. Drug Metab Dispos 2022; 50:1119-1125.

[11] Benoit-Biancamano MO, Connelly J, Villeneuve L *et al.* Deferiprone glucuronidation by human tissues and recombinant UDP glucuronosyltransferase 1A6: an in vitro investigation of genetic and splice variants. Drug Metab Dispos 2009; 37:322-329.

[12] Milne AM, Burchell B, Coughtrie MW. A novel method for the immunoquantification of UDP-glucuronosyltransferases in human tissue. Drug Metab Dispos 2011; 39:2258-2263.

[13] Margaillan G, Rouleau M, Fallon JK *et al.* Quantitative profiling of human renal UDP-glucuronosyltransferases and glucuronidation activity: a comparison of normal and tumoral kidney tissues. Drug Metab Dispos 2015; 43:611-619.

[14] Knights KM, Spencer SM, Fallon JK *et al.* Scaling factors for the in vitro-in vivo extrapolation (IV-IVE) of renal drug and xenobiotic glucuronidation clearance. Br J Clin Pharmacol 2016; 81:1153-1164.

[15] Kumar S, Samuel K, Subramanian R *et al.* Extrapolation of diclofenac clearance from in vitro microsomal metabolism data: role of acyl glucuronidation and sequential oxidative metabolism of the acyl glucuronide. J Pharmacol Exp Ther 2002; 303:969-978.

[16] Carlile DJ, Hakooz N, Bayliss MK, Houston JB. Microsomal prediction of in vivo clearance of CYP2C9 substrates in humans. Br J Clin Pharmacol 1999; 47:625-635.

[17] Yasar U, Eliasson E, Forslund-Bergengren C *et al.* The role of CYP2C9 genotype in the metabolism of diclofenac in vivo and in vitro. Eur J Clin Pharmacol 2001; 57:729-735.

[18] Galetin A, Houston JB. Intestinal and hepatic metabolic activity of five cytochrome P450 enzymes: impact on prediction of first-pass metabolism. J Pharmacol Exp Ther 2006; 318:1220-1229.

[19] Hayley S. Brown AG, David Hallifax and J. Brian Houston. Prediction of In Vivo Drug-DrugInteractions from In Vitro Data.

[20] Ghosal A, Hapangama N, Yuan Y *et al.* Identification of human UDP-glucuronosyltransferase enzyme(s) responsible for the glucuronidation of ezetimibe (Zetia). Drug Metab Dispos 2004; 32:314-320.

[21] Soars MG, Ring BJ, Wrighton SA. The effect of incubation conditions on the enzyme kinetics of udp-glucuronosyltransferases. Drug Metab Dispos 2003; 31:762-767.

[22] Mano Y, Usui T, Kamimura H. The UDP-glucuronosyltransferase 2B7 isozyme is responsible for gemfibrozil glucuronidation in the human liver. Drug Metab Dispos 2007; 35:2040-2044.

[23] Zhang H, Basit A, Busch D *et al.* Quantitative characterization of UDP-glucuronosyltransferase 2B17 in human liver and intestine and its role in testosterone first-pass metabolism. Biochem Pharmacol 2018; 156:32-42.

[24] Ohtsuki S, Schaefer O, Kawakami H *et al.* Simultaneous absolute protein quantification of transporters, cytochromes P450, and UDP-glucuronosyltransferases as a novel approach for the characterization of individual human liver: comparison with mRNA levels and activities. Drug Metab Dispos 2012; 40:83-92.

[25] Achour B, Russell MR, Barber J, Rostami-Hodjegan A. Simultaneous quantification of the abundance of several cytochrome P450 and uridine 5'-diphospho-glucuronosyltransferase enzymes in human liver microsomes using multiplexed targeted proteomics. Drug Metab Dispos 2014; 42:500-510.

[26] Zhang HF, Wang HH, Gao N *et al.* Physiological Content and Intrinsic Activities of 10 Cytochrome P450 Isoforms in Human Normal Liver Microsomes. J Pharmacol Exp Ther 2016; 358:83-93.

[27] Couto N, Al-Majdoub ZM, Achour B *et al.* Quantification of Proteins Involved in Drug Metabolism and Disposition in the Human Liver Using Label-Free Global Proteomics. Mol Pharm 2019; 16:632-647.

[28] Prasad B, Bhatt DK, Johnson K *et al.* Abundance of Phase 1 and 2 Drug-Metabolizing Enzymes in Alcoholic and Hepatitis C Cirrhotic Livers: A Quantitative Targeted Proteomics Study. Drug Metab Dispos 2018; 46:943-952.

[29] Yang J, Jamei M, Yeo KR *et al.* Prediction of intestinal first-pass drug metabolism. Curr Drug Metab 2007; 8:676-684.

[30] Mizuma T. Intestinal glucuronidation metabolism may have a greater impact on oral bioavailability than hepatic glucuronidation metabolism in humans: a study with raloxifene, substrate for UGT1A1, 1A8, 1A9, and 1A10. Int J Pharm 2009; 378:140-141.

[31] Cubitt HE, Houston JB, Galetin A. Relative importance of intestinal and hepatic glucuronidation-impact on the prediction of drug clearance. Pharm Res 2009; 26:1073-1083.

[32] Varma MV, Obach RS, Rotter C *et al.* Physicochemical space for optimum oral bioavailability: contribution of human intestinal absorption and first-pass elimination. J Med Chem 2010; 53:1098-1108.

[33] Boulton DW, Kasichayanula S, Keung CF *et al.* Simultaneous oral therapeutic and intravenous (1)(4)C-microdoses to determine the absolute oral bioavailability of saxagliptin and dapagliflozin. Br J Clin Pharmacol 2013; 75:763-768.

[34] Raje S, Callegari E, Sahasrabudhe V *et al.* Novel Application of the Two-Period Microtracer Approach to Determine Absolute Oral Bioavailability and Fraction Absorbed of Ertugliflozin. Clin Transl Sci 2018; 11:405-411.

[35] Nishimuta H, Sato K, Yabuki M, Komuro S. Prediction of the intestinal first-pass metabolism of CYP3A and UGT substrates in humans from in vitro data. Drug Metab Pharmacokinet 2011; 26:592-601.
